# Methods for Mitochondrial DNA Damage and Depletion in Immortalized Trabecular Meshwork Cells

**DOI:** 10.3390/ijms26136255

**Published:** 2025-06-28

**Authors:** Shane P. Kennedy, Emily Tsaturian, Linlin Zhao, Joshua T. Morgan

**Affiliations:** 1 Department of Cell, Molecular, and Developmental Biology, University of California, Riverside, CA 92521, USA; 2 Department of Bioengineering, University of California, Riverside, CA 92521, USA; 3 Department of Chemistry, University of California, Riverside, CA 92521, USA

**Keywords:** glaucoma, mitochondria, trabecular meshwork

## Abstract

Mitochondrial DNA (mtDNA) damage in trabecular meshwork (TM) cells occurs in open-angle glaucoma (OAG). However, current in vitro models for OAG-like changes in TM cells do not explicitly incorporate mtDNA damage. This work validated two methods of mtDNA damage in immortalized TM cells and assessed OAG-associated expression changes. mtDNA was depleted in TM-1 cells via both ethidium bromide (EtBr) treatment and doxycycline (Dox) induction of a mutant (Y147A) version of Uracil DNA Glycosylase 1 (UNG1) in TM-1 cells (TM-1^rtTAadv-TRE-UNG1Y147A^). Levels of mitochondrial proteins (ATP5F1A, COXII, and COXIV) were measured via western blot. mtDNA levels and mRNA for OAG-associated transcripts (*CTGF*, *FN1*, *PAI1*, and *SFRP1*) were measured by qPCR. There was a statistically significant decrease in mtDNA levels per cell at all treatment times in both EtBr-treated TM-1 cells and induced TM-1^rtTAadv-TRE-UNG1Y147A^ cells. Protein levels of ATP5F1A were not significantly changed; COXII and COXIV showed significant decreases after both EtBr and Dox induction. Both models resulted in upregulation of *CTGF*, *FN1*, and *PAI1*; additionally, EtBr treatment but not Dox induction resulted in *SFRP1* upregulation. In conclusion, two models of mitochondrial depletion were demonstrated in immortalized TM cells; damage was associated with increases in OAG-associated transcripts, supporting a link between mitochondrial damage and glaucoma phenotypes.

## 1. Introduction

Open-angle glaucoma (OAG) is the second leading cause of irreversible blindness worldwide [1]; major subtypes include Pseudo-Exfoliative Glaucoma (PEXG) and Primary OAG (POAG) [2,3]. In both PEXG and POAG, changes in the trabecular meshwork (TM) restrict the outflow of aqueous humor, leading to increases in intraocular pressure (IOP) and disease progression [4]. Substantial research has been dedicated to identifying OAG-associated changes to the TM; of note, fibrosis [4,5], TM cell viability [6,7], and oxidative stress [8,9] are associated with the disease. However, this progress has faced challenges in clinical translation, with only one approved therapy targeting the TM [10]. There is a need to elucidate additional OAG-associated mechanisms in the TM to identify novel therapeutic targets. Prior research has identified mitochondrial damage and dysregulation in TM aging and OAG. Most notably, mitochondrial DNA (mtDNA) has been shown to exhibit oxidative damage (demonstrated by the presence of 8-oxo-7,8-dihydro-2′-deoxyguanosine), the mitochondrial common deletion, mtDNA mutations, and changes in mtDNA per cell [11,12,13,14]. Further, there is evidence of disruption of the electron transport chain in POAG, especially Complex I [15]; importantly, disruption of Complex I itself can lead to upregulation of reactive oxygen species (ROS), further damaging the cell. Despite these promising findings, the role of mitochondrial dysfunction in OAG remains poorly elucidated.

Mitochondria perform many functions in the cell. Most prominently, they generate ATP through electron transport chain-mediated membrane potential but have numerous other regulatory roles, including regulating cellular ROS [16]. The proteins required for mitochondrial function are encoded in part by nuclear DNA and in part by mitochondrial DNA (mtDNA). All of the protein complexes involved in the electron transport chain have at least one subunit encoded by mtDNA and several proteins encoded by nuclear DNA. While a complete description of the electron transport chain is outside the scope of this paper, three gene products are especially relevant for this study. ATP synthase F1 subunit alpha (ATP5F1A), a nuclear-encoded subunit of ATP synthase, catalyzes the synthesis of ATP. Cytochrome c oxidase subunit II (COXII), an mtDNA encoded gene, is involved in the transfer of electrons in the final stages of the electron transport chain. Cytochrome c oxidase subunit IV (COXIV) is also a catalyst in the electron transport chain, playing a similar role to COXII; however, it is encoded by nuclear DNA.

Replication of the mitochondrial genome and synthesis of additional mitochondrial proteins is primarily regulated by two transcription factors. One is Peroxisome Proliferator-Activated Receptor-γ Coactivator-1α (PGC-1α), often referred to as the master regulator of mitochondrial biogenesis [17,18,19,20,21]. This activates a series of other factors, including nuclear respiratory factors, which trigger the synthesis of mitochondrial proteins [22,23]. One of these factors, mitochondrial transcription factor A (TFAM), activates transcription of the mitochondrial genome [24,25]. In addition, TFAM plays a role in packaging [26,27,28] and degrading [29] mtRNA. PGC-1α is also known to respond to damage by ROS via the expression of antioxidant enzymes [30]. One such antioxidant is superoxide dismutase, which catalyzes the conversion of superoxide anions (O_2_^−^) to hydrogen peroxide (H_2_O_2_) [31]. Another related antioxidant enzyme involved is catalase, which catalyzes the degradation of H_2_O_2_ into water and oxygen [32]. Catalase has been known to be active in the calf trabecular meshwork [33], and single-nucleotide polymorphisms in the catalase gene have been linked to primary open-angle glaucoma in Chinese populations [34].

The above data highly suggest that there is a link between glaucoma and mitochondrial dysfunction, but the mechanisms (and their directionality) of this link remain poorly understood. Prior work has demonstrated that mitochondrial function, specifically ATP generation, is dysregulated in TM cells treated with dexamethasone in vitro, demonstrating that glaucoma stimuli can disrupt mitochondrial function [35,36,37]. However, it remains unknown if mitochondrial damage can contribute to glaucoma progression. Here, we turn to prior work in other cell and tissue types to identify potential models. One potential model is treatment with doses of ethidium bromide (EtBr) between 10 ng/mL and 1 µg/mL, which can be an effective way of inducing mitochondrial DNA damage and depletion in several cell types [38,39,40,41,42,43]. It is believed that EtBr limits mtDNA synthesis and can trigger damage through excessive supercoiling and dsDNA breaks in the circular mtDNA [40,43], leading to the degradation of mtDNA [44]. Importantly, the effects of EtBr on nuclear DNA are more limited [40,41,45]. EtBr has minor influences on nuclear DNA levels at higher concentrations between 1 µg/mL and 5 µg/mL, while mtDNA is significantly inhibited at lower concentrations [40,45]. Another potential model is the disruption of the mtDNA repair system. Human Uracil DNA Glycosylase is a base excision DNA repair enzyme that removes uracil that has been incorporated into DNA; human Uracil DNA Glycosylase 1 (UNG1) operates specifically in the mitochondria. A mutant version of this enzyme with tyrosine substituted by alanine at amino acid position 147 (UNG1^Y147A^) removes thymine in addition to uracil, resulting in damage to mtDNA [46]. This has been engineered into a tetracycline-inducible expression vector with an MYC tag, providing a useful tool for inducible mtDNA damage [47]. When induced by tetracycline or doxycycline (Dox) treatment, expressing cells produce UNG1^Y147A^, leading to temporally controlled mtDNA depletion. Importantly, mtDNA depletion occurs within hours, but defects in mitochondrial function can take up to 6 d to establish [47]. The mechanism(s) by which this mtDNA depletion occurs is still unclear, but there is strong evidence to suggest that the depletion observed with this model is the result of active degradation rather than a simple inhibition of replication [47]. In particular, it is believed that the mtDNA is degraded via the formation of abasic sites, which are known to lead to mtDNA degradation by TFAM [29]. In summary, EtBr and UNG1^Y147A^ may provide accessible in vitro approaches for modeling mitochondrial damage in OAG.

Glaucoma is a disease with a complex etiology. While several genetic links have been identified, most notably myocilin (*MYOC*), none are fully predictive of disease onset. For example, mutations in MYOC are associated with approximately 5% of adult-onset OAG cases [48]. However, several phenotypes are well preserved. As mentioned above, fibrotic changes in the TM are broadly associated with disease progression in POAG and PEXG [4,5]. While not exhaustive, several markers are associated with this phenotype. These include Connective Tissue Growth Factor (CTGF), Fibronectin (FN1), and Plasminogen Activator Inhibitor 1 (PAI1). CTGF is a signaling protein that is involved in the regulation of extracellular matrix proteins [49]. Elevated levels of CTGF have been found in the aqueous humor (AH) of patients with PEXG [50], and CTGF has been directly linked to the expression of glaucoma-related ECM proteins [50,51]. Further, elevation of CTGF to the anterior chamber of the mouse eye leads to increased IOP and optic nerve damage [51]. FN1 is a major extracellular matrix (ECM) protein and is significantly increased in the TM in both normal aging and POAG [52]. Furthermore, even though FN1 has yet to be causatively linked to increased IOP, an in vitro study has shown that HTM monolayers have reduced permeability when treated with FN1 [53]. Although increased ECM production is involved in OAG, including FN1, decreased ECM removal may also play a role. Inhibition of matrix metalloproteases (MMPs) has been shown to inhibit aqueous humor outflow through the TM in ex vivo experiments [54,55]. Elevated levels of PAI1, a protease inhibitor, have been found in the aqueous humor of patients with glaucoma, including POAG [56]. Further, PAI1 has been mechanistically linked to MMP inhibition in TGF-β2 induced TM cells in vitro [57].

Additionally, inhibition of the Wnt signaling pathway through Secreted Frizzled-Related Protein 1 (SFRP1) has been linked to OAG. In particular, one study showed that *SFRP1* mRNA is elevated 4.5 fold in glaucomatous cultured human trabecular meshwork cells, coupled with a 50% increase in SFRP1 protein levels [58]. Furthermore, administration of SFPR1 in both an in vivo mouse model and an ex vivo human perfusion model resulted in increased IOP [58]. While the mechanism is unclear, SFRP1 expression in TM cells is elevated in response to ECM stiffness [59] and fibrotic stimuli of TGF-β2 and dexamethasone [60,61]; further, exogenous SFRP1 itself can increase TM stiffness [62].

Here, we show the first demonstration of in vitro mitochondrial depletion in human TM cells using both EtBr and UNG1^Y147A^ in an immortalized cell model. We demonstrate the loss of mtDNA using both methods. This loss of mtDNA is associated with a loss of expression in proteins (COXII and COXIV) associated with mitochondrial function. Importantly, both models led to the upregulation of OAG-associated transcripts *FN1*, *CTGF*, and *PAI1*, while EtBr treatment also resulted in increased *SFRP1* expression. These data suggest a mechanistic link between the mtDNA damage in TM and known markers of OAG. These models may provide useful tools for future studies assessing the role of mitochondrial dysfunction in OAG.

## 2. Results

### 2.1. Ethidium Bromide Treatment Depletes mtDNA

**Figure 1:**
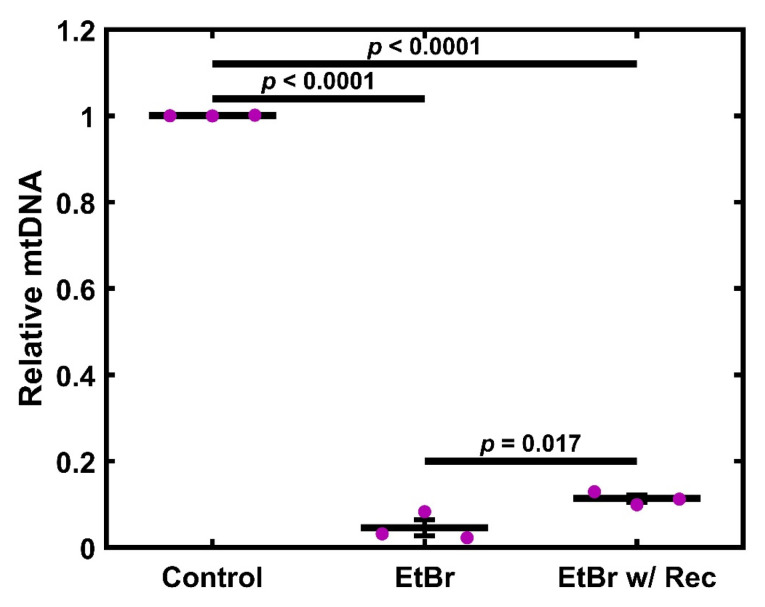
EtBr depletes mtDNA in TM-1 cells. Treatment with 50 ng/mL EtBr for 6 d, as well as EtBr treatment for 4 d with 2 d recovery, resulted in a significant decrease in mtDNA levels compared to the control. Additionally, although overall levels remained low, the recovery case had significantly increased mtDNA compared to the full 6 d treatment. Magenta ● represent individual experiments; mean and standard deviation error bars are represented by black lines. Control data normalized to 1 is shown for reference. Significance between the two groups is indicated by horizontal lines and the stated *p* values, assessed by ANOVA followed by Tukey’s post hoc.

TM-1 cells were treated with 50 ng/mL EtBr for 6 d or treated for 4 d and allowed to recover for 2 d; data were normalized to the experimental control. Compared to control, both treatment groups showed a significant decrease in mtDNA levels (*p* < 0.0001, Figure 1). Additionally, the recovery group was significantly increased compared to the full treatment (*p* = 0.017). We additionally tested shorter time courses, observing very similar results. Moreover, 50 ng/mL EtBr for 4 d is also significantly decreased compared to the control (Figure S1). As a preliminary demonstration of a similar effect in primary cells, we measured mtDNA levels in EtBr-treated primary human TM (HTM) cells. At 6 d, primary HTM cells showed decreased mtDNA levels after both 50 and 75 ng/mL (Figure S2A). Further, 50 ng/mL EtBr induced an apparent reduction in mtDNA, which was apparent at 2 d, 4 d, 6 d, and after 4 d treatment and 2 d recovery (Figure S2B).

### 2.2. mtDNA Depletion Is Coupled to Loss of Cytochrome C Oxidase but Not ATP Synthase

**Figure 2:**
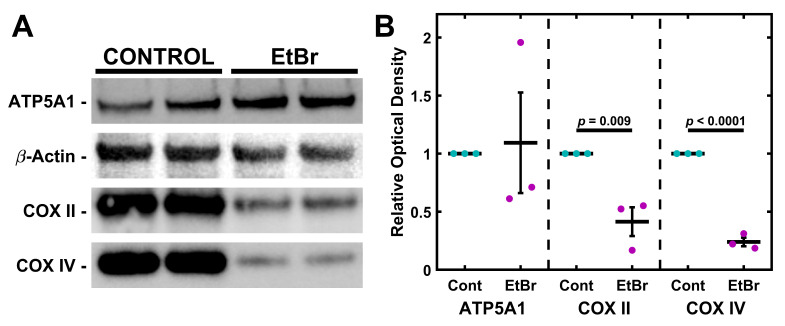
EtBr depletes subunits of Cytochrome C Oxidase but not ATP Synthase. (**A**) Treatment with 50 ng/mL EtBr for 6 d results in decreased expression of COXII and COXIV but not ATP5A1. (**B**) Quantification of triplicate Western blots. Magenta/cyan ● represent individual control/EtBr experiments, respectively; mean and standard deviation error bars are represented by black lines. Control data normalized to 1 is shown for reference. Significance between the two groups is indicated by horizontal lines and the stated *p* values, assessed by *t*-test.

TM-1 cells were treated with 50 ng/mL EtBr for 6 d. Protein was then isolated and analyzed via Western blot for semi-quantitative expression levels of ATP5A1, COXII, and COXIV (Figure 2). Overall, there was no significant change in ATP5A1, but the Cytochrome C Oxidase subunits COXII and COXIV significantly declined. Similar findings were seen with 4 d treatments (Figure S3). We further extracted mRNA from TM-1 cells treated with 50 ng/mL EtBr for 6 d and assayed the transcript expression of mitochondrial regulators and antioxidant enzymes (Figure S4). *PGC1A* and *TFAM* were both significantly reduced (*p* < 0.0001 and *p* = 0.017, respectively). *SOD2* expression was similarly reduced (*p* = 0.025), while there was no significant change to *CATA* expression (*p* = 0.941).

### 2.3. EtBr Treatment Increases Glaucoma-Associated Gene Expression

**Figure 3:**
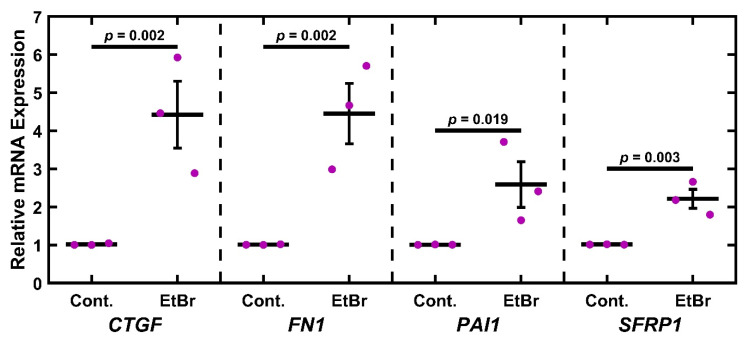
EtBr treatment upregulates glaucoma-associated genes. Treatment with 50 ng/mL EtBr for 6 d results in significantly increased expression in *CTGF*, *FN1*, *PAI1,* and *SFRP1*. Magenta ● represent individual experiments; mean and standard deviation error bars are represented by black lines. Control data normalized to 1 is shown for reference. Significance between the two groups is indicated by horizontal lines and the stated *p* values, assessed by *t*-test on log-transformed values.

Having established that EtBr treatment depleted mitochondria and components of the electron transport chain, we wished to establish if this damage was associated with a glaucomatous phenotype. To this end, TM-1 cells were treated with 50 ng/mL EtBr for 6 d and assayed for glaucoma-associated transcripts (*CTGF*, *FN1*, *PAI1*, and *SFRP1*) via qPCR and compared to controls via paired-sample t-test (Figure 3). There was a significant increase in levels of mRNA coding for *CTGF* (*p* = 0.002), *FN1* (*p* = 0.002), *PAI1* (*p* = 0.019), and *SFRP1* (*p* = 0.003). As a preliminary demonstration of a similar effect in primary cells, we measured mRNA levels in EtBr-treated primary HTM cells (Figure S2C). We observed that 50 ng/mL EtBr treatment induced an apparent increase in *CTGF*, *FN1*, *PAI1*, and *SFRP1* in primary HTM cells, although this was not as robust as in the immortalized TM-1 (Figure 3).

### 2.4. UNG1^Y147A^ Expression in TM-1 Cells Depletes mtDNA

**Figure 4:**
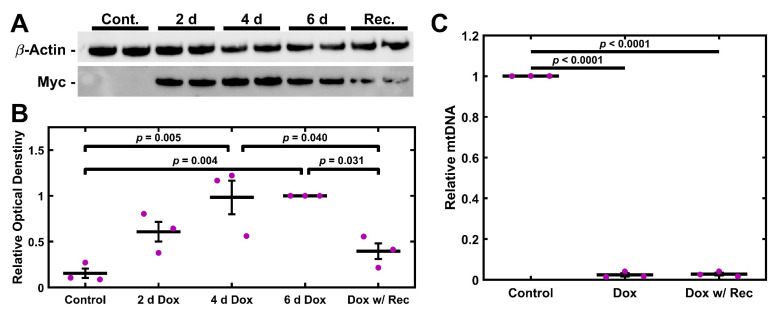
UNG1^Y147A^ Expression depletes mtDNA in TM-1 cells. (**A**) Treatment with 3.5 µg/mL Dox results in detectable expression of UNG1^Y147A^ in TM-1^rtTAadv-TRE-UNG1Y147A^ cells at multiple time points, assayed by the protein’s Myc tag. (**B**) When normalized to the 6 d Dox treatment, UNG1^Y147A^ was significantly elevated at 4 d and 6 d compared to the control. Further, the 4 d Dox with 2 d recovery resulted in significantly decreased UNG1^Y147A^ expression compared to 4 d and 6 d. (**C**) Treatment with 3.5 µg/mL Dox for 6 d, as well as Dox treatment for 4 d with 2 d recovery, resulted in a significant decrease in mtDNA levels compared to the control. Magenta ● represent individual experiments; mean and standard deviation error bars are represented by black lines. Control data normalized to 1 is shown for reference. Significance between the two groups is indicated by horizontal lines and the stated *p* values, assessed by ANOVA followed by Tukey’s post hoc.

Having established a chemical model of mitochondrial depletion in TM cells, we wished to establish an alternative genetic method. TM-1 cells were double transduced to express rtTA-Advanced (rtTAadv) and UNG1^Y147A^ under a tetracycline-responsive promoter (TRE-UNG^Y147A^), allowing for Dox-inducible expression of UNG1^Y147A^. We denote these cells as TM-1^rtTAadv-TRE-UNG1Y147A^ cells; they were treated with 3.5 µg/mL Dox, and the expression of UNG1^Y147A^ was assessed via western blotting for the Myc tag. Expression was detectable at 2, 4, and 6 d of 3.5 µg/mL Dox treatment and was additionally detectable after 4 d Dox and 2 d recovery (Figure 4A). Myc was not readily detectable in a 6 d control culture. When semi-quantitatively assessed using relative optical density compared to the 6 d treatment, both 4 d and 6 d treatments were significantly elevated compared to the 6 d control (*p* = 0.005 and *p* = 0.004, respectively; Figure 4B). Further, there was significantly decreased expression in the recovery condition when compared to both 4 d and 6 d (*p* = 0.040 and *p* = 0.031, respectively). UNG1^Y147A^ expression was accompanied by loss of mtDNA. TM-1^rtTAadv-TRE-UNG1Y147A^ cells were treated with 3.5 µg/mL Dox for 6 d or treated 4 d and allowed to recover for 2 d; data was normalized to the 6 d control culture of TM-1^rtTAadv-TRE-UNG1Y147A^. Compared to the control, both treatment groups showed a significant decrease in mtDNA levels (*p* < 0.001, Figure 4C); however, there was no significant difference between the two treatments. Similar results were observed with the 4 d treatment when compared to the same 6 d control (Figure S5).

### 2.5. UNG1^Y147A^ Meditated mtDNA Depletion Correlates to Loss of Cytochrome C Oxidase but Not ATP Synthase

**Figure 5:**
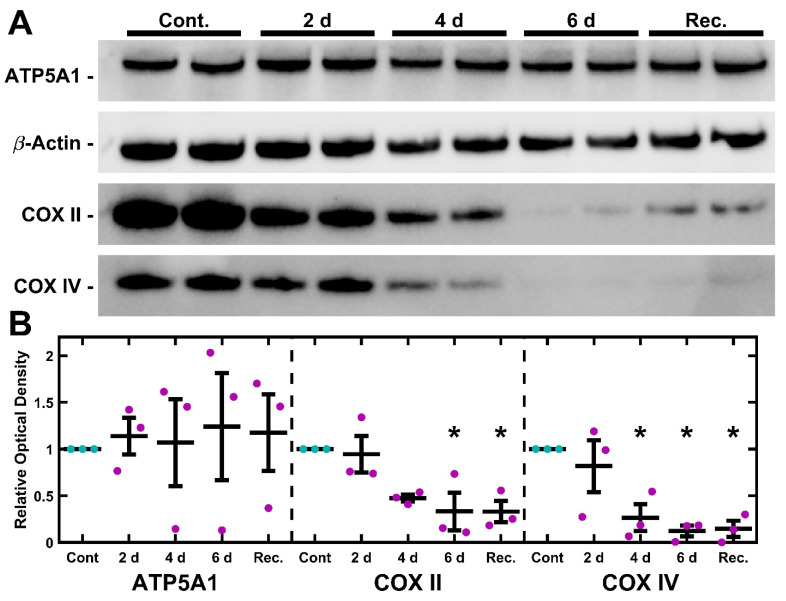
UNG1^Y147A^ depletes Cytochrome C Oxidase subunits in TM-1 cells. (**A**) Representative blots of ATP5A1, COXII, and COXIV are shown, and the paired loading control β-actin blot is repeated from Figure 4. Dox induction resulted in a decline in COXII and COXIV but minimal effect in ATP5A1 expression. (**B**) Quantification of the optical density revealed no significant changes to ATP5A1. When compared to the control, COXII expression was significantly decreased after 6 d Dox treatment, as well as 4 d Dox followed by 2 d recovery. Similarly, COXIV was also significantly reduced at 4 d, 6 d, and the recovery condition. Magenta/cyan ● represent individual control/Dox experiments, respectively; mean and standard deviation error bars are represented by black lines. Control data normalized to 1 is shown for reference. Significance in reference to the 6 d control was assessed by ANOVA followed by Dunnett’s post hoc test. * indicates *p* < 0.05; *p*-values provided in the main text.

TM-1^rtTAadv-TRE-UNG1Y147A^ cells were treated with 3.5 µg/mL Dox for 2, 4, and 6 d to induce the expression of UNG1^Y147A^. In addition, a fourth treatment was carried out whereby cells were treated with 3.5 µg/mL Dox for 4 d and then were left untreated for an additional 2 d (recovery). Untreated 6 d cultures were used as the control. Protein was then isolated and analyzed via Western blot for semi-quantitative expression levels of ATP5A1, COXII, and COXIV (Figure 5). Optical density compared to loading control was used to determine levels of each protein, normalized to 6 d control culture. Relative optical density was assessed using one-way ANOVA followed by Dunnett’s post hoc test. There was a statistically significant decrease in COXII at both 6 d and recovery (*p* = 0.020 and *p* = 0.020, respectively; Figure 5B). Further, there was a statistically significant decrease in COXIV at 4 d, 6 d, and recovery (*p* = 0.018, *p* = 0.006, and *p* = 0.007, respectively).

### 2.6. UNG1^Y147A^ Alters Glaucoma-Associated Gene Expression

**Figure 6:**
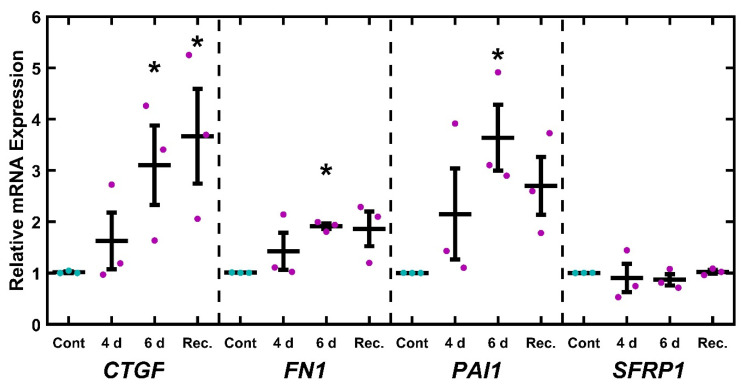
Glaucoma-related mRNA transcripts response to mtDNA depletion. After 6 d Dox induction, TM-1^rtTAadv-TRE-UNG1Y147A^ cells showed a significant increase in *CTGF*, *FN1*, and *PAI1* expression. Additionally, CTGF was significantly upregulated after 4 d treatment with 2 d recovery. Magenta/cyan ● represent individual control/Dox experiments, respectively; mean and standard deviation error bars are represented by black lines. Control data normalized to 1 is shown for reference. Significance in reference to the 6 d control was assessed by ANOVA followed by Dunnett’s post hoc test. * indicates *p* < 0.05; *p*-values provided in the main text.

Similar to the EtBr studies, we wished to assess if inducing mitochondrial damage correlates with altered expression of glaucoma-related transcripts. TM-1^rtTAadv-TRE-UNG1Y147A^ cells were treated with 3.5 µg/mL Dox for 4 and 6 d to induce expression of UNG1^Y147A^. In addition, an additional treatment was carried out whereby cells were treated with 3.5 µg/mL Dox for 4 d and then left untreated for an additional 2 d (recovery). The expression of glaucoma-associated transcripts (*CTGF*, *FN1*, *PAI1*, and *SFRP1*) was measured via qPCR and assessed using one-way ANOVA followed by Dunnett’s post hoc testing on the log-transformed values (Figure 6). *CTGF* was significantly upregulated at 6 d and recovery (*p* = 0.049 and *p* = 0.024, respectively). Similarly, *FN1* and *PAI1* were significantly upregulated at the 6 d time point (*p* = 0.048 and *p* = 0.014, respectively). No significant changes were observed with *SFRP1* expression. As Dox has previously been reported to alter ECM-related gene expression [63,64], we similarly treated untransduced TM-1 cells (e.g., lacking rtTAadv and UNG1^Y147A^) with Dox and found no significant effects on the expression of *CTGF*, *FN1*, *PAI1*, and *SFRP1* (Figure S6).

## 3. Discussion

In this paper, we demonstrated two complementary models of mtDNA depletion in TM-1 cells via treatment with EtBr and via Dox induction of TM-1^rtTAadv-TRE-UNG1Y147A^. We further showed decreases in the level of mitochondrial electron transport chain subunits that are required to maintain mitochondrial membrane polarization and changes in the expression of glaucoma-associated transcripts. To our knowledge, these are the first in vitro TM studies to directly damage mtDNA and assess changes in glaucoma-related gene expression, offering a potential tool to study the mechanistic role of previously reported mitochondrial dysfunction in POAG and PEXG [11,12,13,14].

Both models strongly disrupt mitochondria, shown most evidently by the loss of mtDNA (Figure 1 and Figure 4). Importantly, impacts on electron transport chain subunits were present in both mitochondrial and nuclear-encoded subunits (Figure 2 and Figure 5). While the loss of COXII may be expected due to a loss of mtDNA copies, decreases in COXIV, which is encoded by nuclear DNA, suggest that these depletion models involve crosstalk between mitochondrial activity and nuclear gene expression. The fact that crosstalk can occur between the mitochondria and nuclear gene expression is well described in other systems [65,66,67], but this has not previously been demonstrated in TM cells. By comparison, the nuclear-encoded ATP5A1 did not show a significant change following either treatment. Mitochondrial damage and depletion could directly contribute to glaucoma pathogenesis through cell loss. It has previously been shown in POAG that there is an increase in apoptosis, cell loss, and senescence of TM cells [68,69,70]. Mitochondria are well understood to be mediators of apoptosis and senescence [71]. In particular, mitochondrial DNA damage has been shown to induce mitochondria-mediated apoptosis [72].

A more indirect mechanism where mitochondrial loss could lead to an increase in glaucoma-related mRNA transcripts is via calcium dysregulation. Mitochondria are heavily involved in calcium regulation [73], and disruption of mitochondria has been linked to disruption in calcium regulation [73,74,75], including in TM cells [15]. Further, dysregulation of transient receptor potential cation channel subfamily V member 4 (TRPV4), a calcium channel, has been implicated in glaucoma [76,77,78,79,80]. For example, one study showed TRPV4 agonists produced a glaucomatous phenotype both in vitro and in vivo, while TRPV4 antagonists were protective [76]; the importance of TRPV4 has been broadly supported by other studies, although the specific mechanisms are unclear [80]. Mitochondrial dysregulation leading to loss of calcium homeostasis could result in similar effects to TRPV4 dysregulation.

OAG can also be characterized by expression changes in the outflow pathway. Both the EtBr and the UNG1^Y147A^ models show a significant increase in transcripts associated with OAG, specifically *CTGF*, *FN1*, and *PAI1* (Figure 3 and Figure 6). However, *SFRP1* was upregulated after EtBr treatment, which was not observed after UNG1^Y147A^ induction. The findings of previously known OAG-associated transcripts in these mtDNA depletion models support further investigation into mitochondrial-mediated OAG mechanisms. While mitochondria do not directly regulate the expression of these genes, we can speculate on potential mechanisms. One promising avenue is the activation of an innate immune response in the cell triggered by damaged mtDNA entering the cytosol of TM cells. A prior study looked at the effects of the presence of mtDNA in the cytosol using mouse embryonic fibroblasts [81]. In this study, cells expressing a mutant TFAM showed a decrease in mtDNA per cell and increasing mtDNA leaking into the cytosol. These were correlated with an increase in the expression of interferon-stimulating genes, typically associated with pathogen response and inflammation [82]. Inflammatory processes are also associated with fibrosis more generally [83,84,85] and increased expression of key OAG-associated transcripts more specifically, including *CTGF* [86], *FN1* [87,88,89], and *PAI1* [90].

Future work elucidating these mechanisms in OAG can take advantage of extensive work performed in the context of mitochondrial dysfunction. OAG is an aging-associated disease, and many of the changes in TM cells in OAG are seen in aging more broadly. For example, the electron transport chain is known to be impaired in POAG, specifically respiratory Complex I [15], possibly due to the presence of the common deletion [11,12]. In addition, one report shows that the generation of ROS from Complex I is associated with shorter lifespans when comparing multiple species [91]. Further, the loss of mtDNA per cell is known to occur in TM cells in POAG and normal aging [12]. This is also a hallmark of aging in many tissues and cells, including the pancreas [92], skeletal muscle [93], adipose tissue [94], and fibroblasts [95]. Another report noted that human skin fibroblasts show an increase in the mitochondrial common deletion with age [96], as do buccal squamous epithelial cells [97], heart cells [98], and skeletal muscle cells [99]. Many other kinds of mitochondrial DNA mutations are known to increase in many tissues with aging as well [95,100,101,102,103].

As with all in vitro models, there are limitations to the described approaches. This work was conducted with an immortalized line (TM-1), which limits the physiological relevance. Primary HTM cells are preferred for in vitro studies, and both EtBr and the lentiviral transduction would be feasible in future primary cell studies. As an initial demonstration that these studies can be adapted to primary HTM, we performed limited studies with EtBr and primary HTM cells (Figure S2). While conclusions cannot be drawn without biological replication, the data demonstrate findings similar to the TM-1 studies, with decreased mtDNA and increased expression of OAG-associated transcripts, although the changes are not as robust as shown in TM-1. Future studies pursuing mechanisms would benefit from validation in primary HTM cells and, additionally, in ex vivo models. Additionally, while the application of these models to TM cells provides compelling data linking mitochondrial dysfunction to glaucoma-related transcription, it is essential to note that this mechanism has not been shown in vivo and may not represent a relevant mechanism of disease progression. It is important to note that we only assessed a limited number of glaucoma-associated targets, and other glaucoma-associated genes, such as *MYOC,* would be valuable to pursue. Further, although both models show a similar pattern in terms of electron transport chain protein complex subunit loss and upregulation of glaucoma-related mRNA transcripts *CTGF*, *FN1*, and *PAI*, there is a notable distinction between the two methods, where *SFRP1* is significantly upregulated by EtBr but not by UNG1^Y147A^ expression. Importantly, the mechanism of these changes has not been elucidated and may not be representative of glaucoma pathogenesis in vivo. Further, as mentioned in the Introduction, EtBr can damage nuclear DNA, although the required dose is expected to be much higher [40,45]; testing of other doses would be relevant. Other off-target effects are expected for both models, and with all models, results should be carefully interpreted. When possible, multiple orthogonal methods should be utilized, possibly in combination, to mitigate the nonspecific effects.

In summary, we present here two new models of a glaucomatous state in immortalized trabecular meshwork cells. These models are novel insofar as they incorporate mtDNA damage and depletion, known to be present in TM cells in both aging and OAG. They also show a decrease in levels of mitochondrial electron transport chain proteins, which may produce an effect not unlike the impairment of COXI, which is known to occur in OAG. The increase in fibrotic mRNA transcripts also suggests a relationship between mitochondrial damage and fibrotic glaucoma pathogenesis. For this reason, these models merit additional study in more physiologically relevant models, including primary human TM cells and ex vivo cultures. While this early work is consistent with prior work associating mitochondrial damage with glaucoma, causative mechanistic links remain poorly understood and are deserving of further study.

## 4. Materials and Methods

### 4.1. Cell Culture

TM-1 cells (gift of Dr. Paul Russell, University of California, Davis) are SV-40 immortalized human TM cells [104,105]; TM-1 cells have known differences when compared to primary HTM cells, including promotor utilization but have been previously used as an accessible cell model for early studies [104,105,106,107,108]. TM-1 cells were routinely cultured in Dulbecco’s modified Eagle medium/Nutrient Mixture F-12 (50:50; DMEM/F12 with L-glutamine and 15 mM HEPES; (Corning, Manassas, VA, USA) supplemented with 10% fetal bovine serum (FBS) and 1% penicillin/streptomycin (P/S) [58]. HEK293TN (System Biosciences, San Jose, CA, USA) were cultured in Dulbecco’s modified Eagle medium with 4.5 g/L glucose, L-glutamine, and sodium pyruvate (Corning, Manassas, VA, USA) supplemented with 10% FBS and 1% P/S.

### 4.2. Plasmid Transfections and Viral Transduction

**Figure 7:**
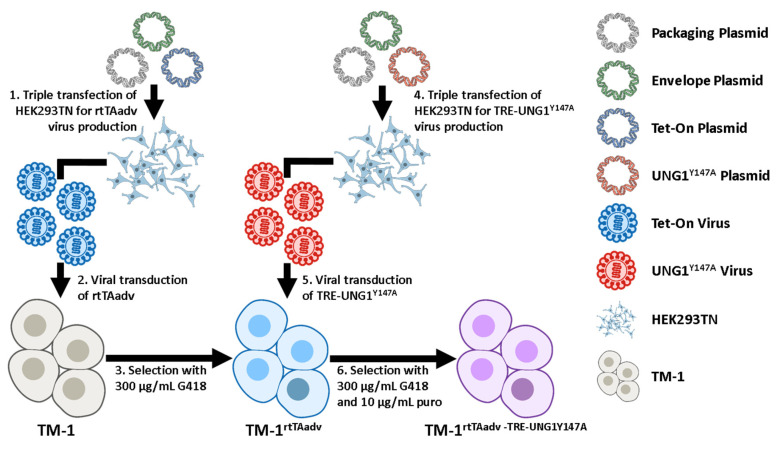
Schematic of the double transduction. (**1**) We performed triple transfection of HEK293TN cells with plasmids for packaging (psPAX2), envelope (pMD2.G), and transfer of the rtTA-Advanced (rtTAadv) construct (pLVX-EF1a-tetOn-IRES-G418 (EtO)). (**2**) Viral supernatant was used to transduce TM-1 cells. (**3**) Transduced cells were selected with G418 to generate a selected population of TM-1^rtTAadv^. (**4**) We performed triple transfection as in (**1**) but with a transfer plasmid for the TRE-UNG1^Y147A^ construct (pMA3287). (**5**) Viral supernatant of the second triple transfection was used to transduce TM1^rtTAadv^ cells. (**6**) Cells were selected under both G418 and puromycin (puro) to generate TM-1^rtTAadv-TRE-UNG1Y147A^ cells. Schematic was constructed with graphics from NIH BIOART [109,110,111,112].

TM-1 cells were sequentially transduced for the expression of the rtTA-Advanced and UNG1^Y147A^ under a Dox-inducible tetracycline response element (TRE)-containing promotor. The approach is described below and shown schematically in Figure 7. For all plasmids used, plasmid maps are accessible from Addgene using the identifiers provided. pLVX-EF1a-tetOn-IRES-G418 (EtO) was a gift from Fred Gage (Addgene plasmid # 84776), pMA3287 was a gift from Mikhail Alexeyev (Addgene plasmid # 46883), and psPAX2 and pMD2.G were gifts from Didier Trono (Addgene plasmid # 12260 and # 12259). Most relevant to this study, pLVX-EF1a-tetOn-IRES-G418 (EtO) confers G418 resistance and enables the constitutive expression of the transcriptional control activator protein rtTA-Advanced (referred to here as rtTAadv), which binds to a TRE and activates genes in the presence of DOX. Further, pMA3287 confers puromycin resistance and has UNG1^Y147A^ under the TRE-containing promotor P_Tight_ (denoted as TRE-UNG1^Y147A^), allowing rtTA-Advanced-mediated expression of UNG1^Y147A^. In combination, they provide the Dox-inducible expression of UNG1^Y147A^. First, to generate an rtTA-Advanced virus, HEK293TN cells were triple transfected with psPAX2, pMD2.G, and the transfer plasmid (pLVX-EF1a-tetOn-IRES-G418 (EtO)) according to manufacturer instructions (TransIT-VirusGEN Transfection Reagent; Mirus Bio LLC, Madison, WI, USA). Media were changed at 24 h, and viral supernatant was harvested at 48 and 72 h. Viral supernatant was centrifuged for 10 min at 1000× *g* and passed through a 0.45 µm filter prior to use. TM-1 cells were then transduced by incubating them with a 1:5 ratio of the rtTAadv viral supernatant and fresh media for 48 h, followed by 48 h in normal media. Viral titer and MOI were not directly measured. Expressing TM-1 cells were selected with 300 µg/mL G418 for 4 days to generate TM-1^rtTAadv^ cells. A second triple transfection was performed to generate the TRE-UNG1^Y147A^ using pMA3287 as a transfer plasmid. This second virus was used to transduce the previously transduced TM-1^rtTAadv^ cells, with 10 µg/mL puromycin used for selection in addition to 300 µg/mL G418. The double-transduced TM-1 cells were designated TM-1^rtTAadv-TRE-UNG1Y147A^ and were maintained with 10 µg/mL puromycin and 300 µg/mL G418.

### 4.3. Mitochondrial Depletion Experimental Conditions

For the EtBr model, cells were treated with EtBr (VWR, Radnor, PA, USA) at a dose of 50 ng/mL. Media in both the treated cells and accompanying non-treated controls were changed every two days. We additionally tested 75 ng/mL and found similar but more variable mtDNA depletion (Figure S7). For the UNG1^Y147A^ model, TM-1^rtTAadv-TRE-UNG1Y147A^ cells were treated with 3.5 µg/mL Dox (Doxycycline Hyclate; TCI America, Portland, OR, USA) in water or vehicle control. Untransduced TM-1 cells were treated in parallel as a control for the effects of Dox, which can inhibit mitochondrial protein synthesis [113,114,115]. For both EtBr and UNG1^Y147A^ models, cells were plated at approximately 250,000 cells/well in 6-well plates 24 h prior to treatment initiation and were confluent at treatment. For each experiment and condition, two wells were plated in parallel, one for RNA and DNA and one for protein collection (as described below). All experiments were performed with three biological replicates (three different cell passages plated on separate days). All experiments were performed within 5 passages.

### 4.4. Isolation of DNA and RNA

Cells were treated as described in 4.3 above. In order to determine mtDNA levels per cell, DNA was isolated according to manufacturer instructions (Monarch Genomic DNA Purification Kit; New England Bio Labs, Ipswich, MA, USA). Total RNA was isolated according to manufacturer instructions (ISOLATE II RNA Mini Kit; Meridian Biosciences, Memphis, TN, USA). DNA and RNA concentrations were determined via absorbance using the Spectramax M2 Microplate Reader (Molecular Devices, San Jose, CA, USA).

### 4.5. Quantitative Polymerase Chain Reaction

Quantification of DNA and RNA expression was performed using qPCR according to manufacturer instructions (SensiFAST SYBR No-ROX Kit; Meridian Bioscience, Memphis, TN, USA) on a Magnetic Induction Cycler (Biomolecular Systems, Upper Coomera, Queensland, Australia). Total DNA and RNA concentrations were measured as described in Section 4.4 above. For DNA, 5 ng was loaded per reaction. For RNA, 60 ng was loaded per reaction. TATA-box binding protein (*TBP)* was used as an endogenous control for *CTGF*, *FN1*, *PAI1*, and *SFRP1*. *GAPDH* was used as an endogenous control for *PGC1A*, *TFAM*, *SOD2*, and *CATA*. Microglobin DNA (*BGLOB*) was used as a nuclear reference gene for total mtDNA (TMDQ); change in mtDNA levels was quantified relative to nuclear DNA. All primers are shown in Table 1. All reactions were performed in technical triplicate for each biological replicate.

### 4.6. Protein Isolation and Western Blotting

Cells were treated as described in 4.3 above. Protein was isolated using RIPA buffer (ThermoFisher Scientific, Waltham, MA, USA) in conjunction with Mammalian Protease Inhibitor Cocktail (VWR, Radnor, PA, USA) and was quantified using the DC Protein Assay (Bio-Rad, Hercules, CA, USA). Equivalent protein amounts were separated on NuPAGE 4 to 12% Bis-Tris gels in MES SDS running buffer (ThermoFisher Scientific, Waltham, MA, USA). Protein was transferred to a 0.45 μm nitrocellulose membrane using the Pierce Power Station (Thermo Fisher Scientific, Waltham, MA, USA). Blots were labeled with mouse anti-β-Actin (3700; Cell Signaling Technology, Danvers, MA, USA), mouse anti-GAPDH (60004-1-Ig; Proteintech, Rosemont, IL, USA), mouse anti-Myc (MA1-21316; ThermoFisher), rabbit anti-COXII (55070-1-AP; Proteintech, Rosemont, IL, USA), mouse anti-COXIV (60251-1-Ig; Proteintech), and rabbit anti-ATP5A1 (14676-1-AP; Proteintech). All primary antibodies were used at a 1:500 dilution overnight at 4 °C with rocking. Secondary staining was conducted with HRP-conjugated goat anti-rabbit or goat anti-mouse, as appropriate (SeraCare, Milford, MA, USA). All secondary antibodies were used at a 1:50,000 dilution with 1 h incubation and rocking at room temperature. Blots were developed using Radiance ECL Chemiluminescent HRP substrate (Azure Biosystems, Dublin, CA, USA) and imaged using the ChemiDoc Imaging System (Bio-Rad, Hercules, CA, USA). The relative optical density of bands was determined using ImageJ 1.53m (NIH ImageJ software) and used as a semi-quantitative measure of expression. Target protein band intensity was normalized to either GAPDH or β-Actin. All samples were run in technical duplicates for each biological replicate.

### 4.7. Statistical Analysis

All experiments were conducted in biological triplicate. Data were analyzed via paired-sample t-test in the cases where there was only a control and one experimental group. In experiments with more than one treatment, one-way ANOVA was used with either Tukey’s or Dunnett’s post hoc testing for multiple comparisons. The statistical approach for each experimental set is detailed in the appropriate results section. For mRNA expression analysis, all values were log-transformed prior to testing. All statistical analysis was performed in MATLAB 2022a (Mathworks, Natick, MA, USA). All data used to construct the quantitative figures are provided in Supplemental Data.

## Figures and Tables

**Table 1:** Primers used for qPCR.

Gene	Forward Sequence	Reverse Sequence	Reference
*TBP*	TGTATCCACAGTGAATCTTGGTTG	GGTTCGTGGCTCTCTTATCCTC	[116]
*CTGF*	CACAAGGGCCTCTTCTGTGA	TCTCTTCCAGGTCAGCTTCG	[117]
*FN1*	AATCCAAGCGGAGAGAGTCA	CATCCTCAGGGCTCGAGTAG	[118]
*PAI1*	AATGTGTCATTTCCGGCTGCTGTG	ACATCCATCTTTGTGCCCTACCCT	[119]
*SFRP1*	CTCAACAAGAACTGCCACGC	CTCGTTGTCACAGGGAGGAC	Primer-Blast [120]
*GAPDH*	ACAGTCAGCCGCATCTTCTT	ACGACCAAATCCGTTGACTC	[121]
*PGC1A*	AAACAGCAGCAGAGACAAATGC	TTGGTTTGGCTTGTAAGTGTTGTG	[122]
*TFAM*	TGTTCACAATGGATAGGCAC	TCTGGGTTTTCCAAAGCAAG	[122]
*SOD2*	CTGGACAAACCTCAGCCCT	CTGATTTGGACAAGCAGCAA	[122]
*CATA*	TGGAAAGAAGACTCCCATCG	CCAGAAGTCCCAGACCATGT	[122]
*BGLOB*	GGTGAGTCTATGGGACGCTT	GATCCTGAGACTTCCACACTGA	Primer-Blast [120]
*TMDQ*	CCATCTTTGCAGGCACACTCATC	ATCCACCTCAACTGCCTGCTATG	[11]

## Data Availability

The original contributions presented in this study are included in the article/Supplementary Material. Further inquiries can be directed to the corresponding author(s).

## Supplementary Materials

The following supporting information can be downloaded at https://www.mdpi.com/article/10.3390/ijms26136255/s1.
