# Supplementary material for: Methods for Mitochondrial DNA Damage and Depletion in Immortalized Trabecular Meshwork Cells
Source: Int J Mol Sci. 2025 Jun 28;26(13):6255. doi: 10.3390/ijms26136255 (PMC12250015; doi:10.3390/ijms26136255)
Supplement: Supplementary file 1 [file ijms-26-06255-s001.zip › SK_MitoDep_Supp_s1.pdf]

Supplemental Figures

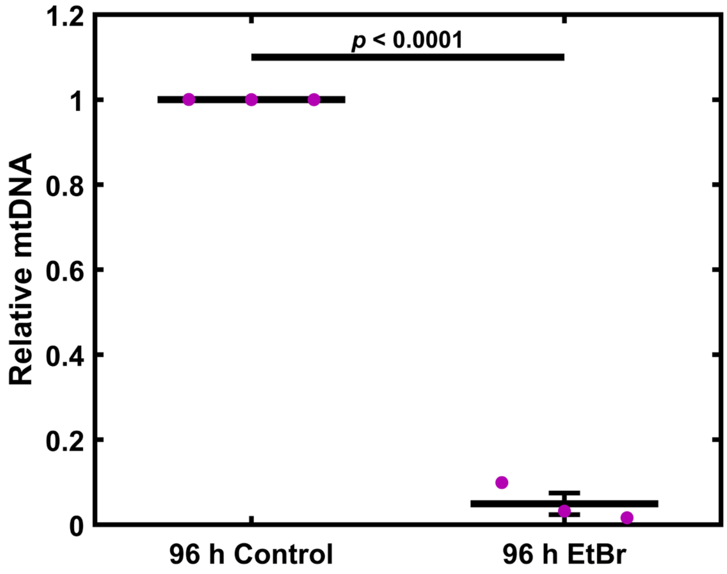

Figure S1: EtBr depletes mtDNA in TM-1 cells. Treatment with 50 ng/mL EtBr for 4 d resulted significant decrease in mtDNA levels compared to 4 d control culture. Magenta ● represent individual experiments; mean and standard deviation error bars are represented by black lines. Significance between two groups is indicated by a horizontal line and the stated *p* value, assessed by paired t-test.

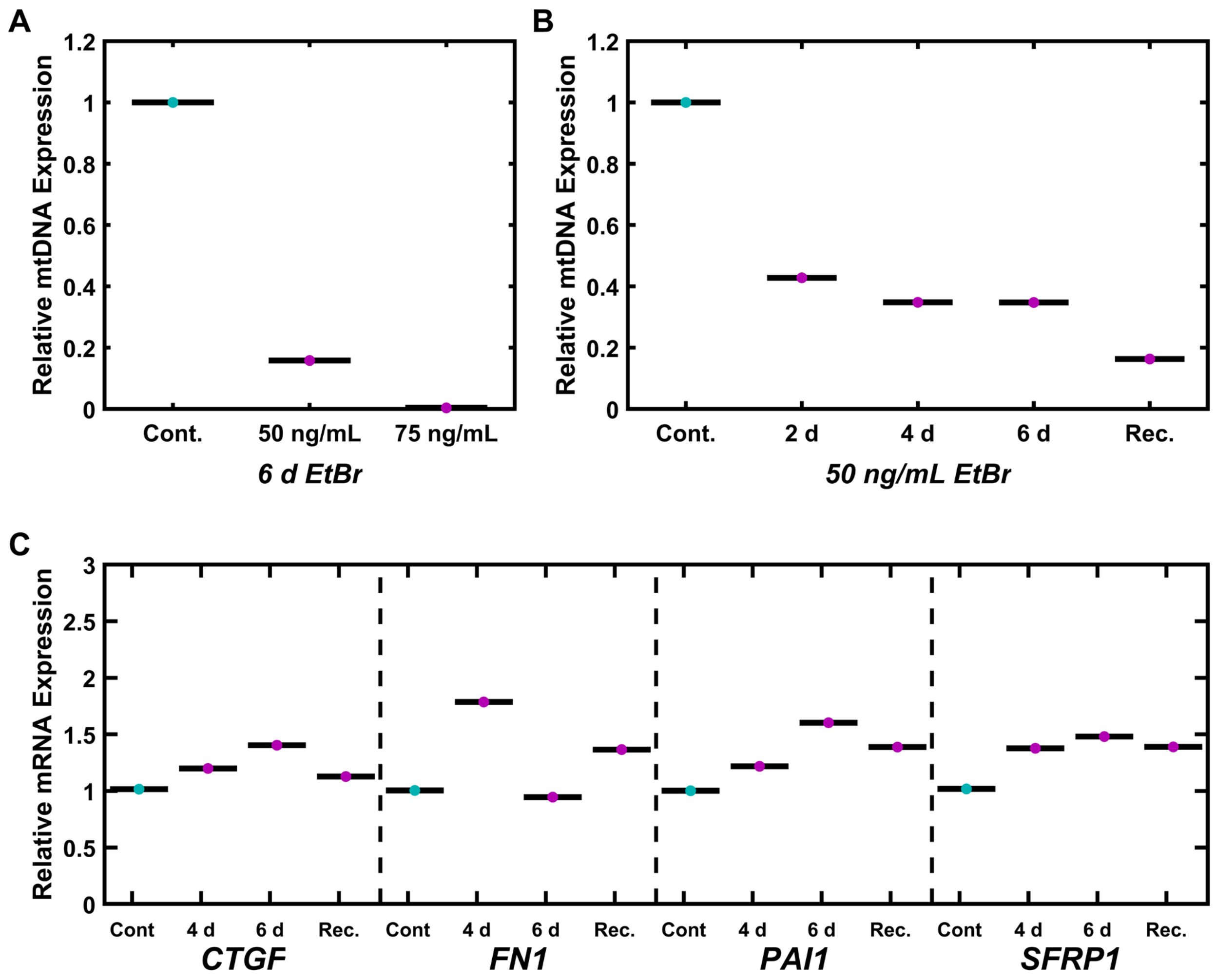

Figure S2: EtBr depletes mtDNA and upregulates glaucoma associated genes in primary HTM cells. All work involving human tissue was performed in a manner consistent with the Declaration of Helsinki. Primary cultures of HTM cells were isolated from a donor human corneoscleral rim deemed unsuitable for transplant (Saving Sight, St. Louis, MO, USA); the researchers had no role in tissue recovery or donor identification. The isolation procedure was performed as we have previously described [37]. All cells were tested as HTM cells via examining myocilin (MYOC) mRNA upregulation after a 3-day 100 nM Dex challenge. Cells used exhibited a ten-fold or greater increase in MYOC mRNA when treated with Dex. Donors were a 71 year old caucasian female (data in panel A) and a 56 year old caucasian male (data in panels B and C). **(A)** Treatment with 50 or 75 ng/mL EtBr for 6 d resulted significant decrease in mtDNA levels

compared to control culture. **(B)** Treatment with 50 ng/mL EtBr for 4 d, 6 d, or 4 d with 2 d recovery (Rec.) resulted in a apparent decreases in mtDNA levels compared to control at all time points. **(C)** Treatment with 50 ng/mL EtBr for 4 d, 6 d, or 4 d with 2 d recovery (Rec.) results in increased expression of target genes. Apparent increases are seen in *CTGF* (4 d, 6 d, and Rec.), *FN1* (4 d and Rec.), *PAI1* (4 d, 6 d, and Rec.) and *SFRP1* (4 d, 6 d, and Rec.). Cyan (control) and Magenta (treated) ● represent individual experiments; mean is represented by black lines (n=1).

References from main text

37. Kennedy, S.; Williams, C.; Tsaturian, E.; Morgan, J.T. Dexamethasone Impairs ATP Production and Mitochondrial Performance in Human Trabecular Meshwork Cells. Curr Issues Mol Biol 2024, 46, 9867–9880, doi:10.3390/cimb46090587

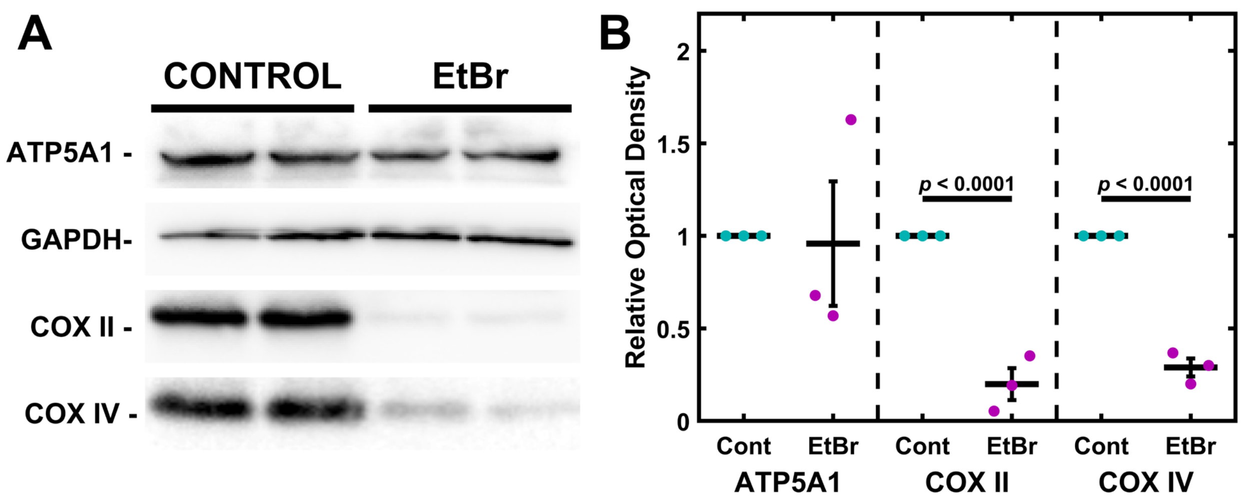

Figure S3: EtBr depletes subunits of Cytochrome C Oxidase but not ATP Synthase. (A) Treatment with 50 ng/mL EtBr for 4 d results in decreased expression of COXII and COXIV, but not ATP5A1. **(B)** Quantification of triplicate Western blots compared to 4 d control cultures. Magenta ● represent individual experiments; mean and standard deviation error bars are represented by black lines. Control data normalized to 1 is shown for reference. Significance between two groups is indicated by horizontal lines and the stated *p* values, assessed by t-test.

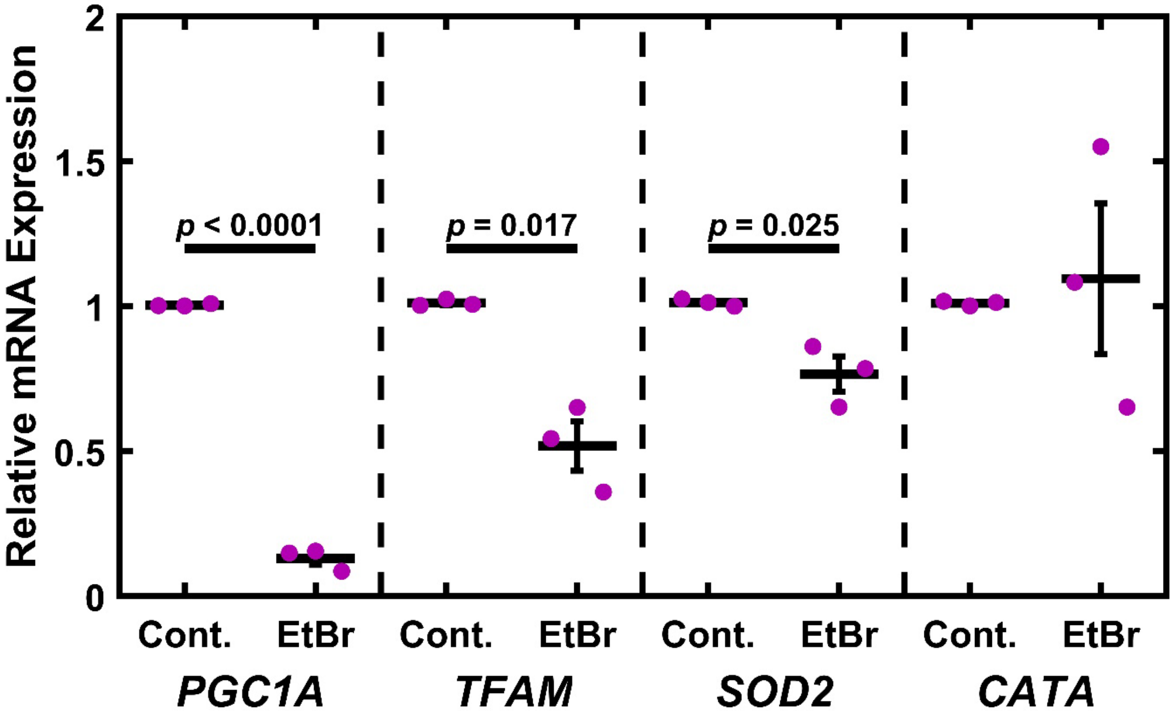

Figure S4: EtBr treatment downregulates mitochondrial and oxidative species associated genes. Treatment with 50 ng/mL EtBr for 6 d results in decreased expression of the mitochondrial regulators *PGC1A* and *TFAM*. *SOD2* expression is similarly reduced, while there is no significant change to *CATA* expression. Magenta ● represent individual experiments; mean and standard deviation error bars are represented by black lines. Control data normalized to 1 is shown for reference. Significance between two groups is indicated by horizontal lines and the stated *p* values, assessed by t-test on log-transformed data.

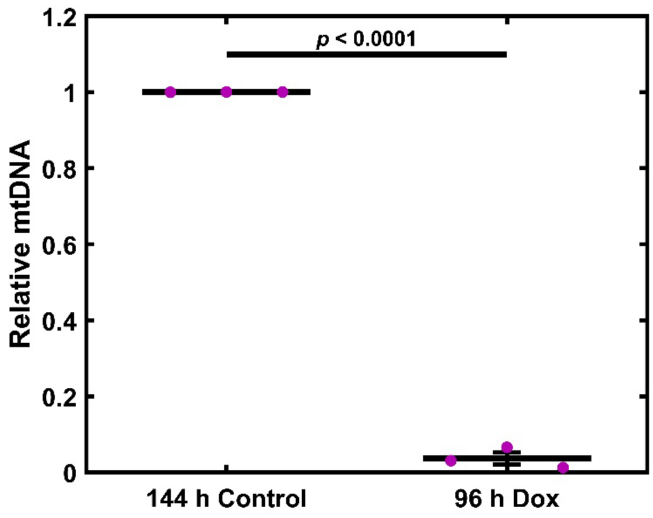

Figure S5: UNG^{Y147A} Expression depletes mtDNA in TM-1 cells. Treatment of TM-1$^{rtTAadv\text{-}TRE\text{-}UNG1Y147A}$ with 3.5 µg/mL Dox for 4 d resulted significant decrease in mtDNA levels compared to a 6 d control culture. Magenta ● represent individual experiments; mean and standard deviation error bars are represented by black lines. Significance between two groups is indicated by a horizontal line and the stated *p* value, assessed by paired t-test.

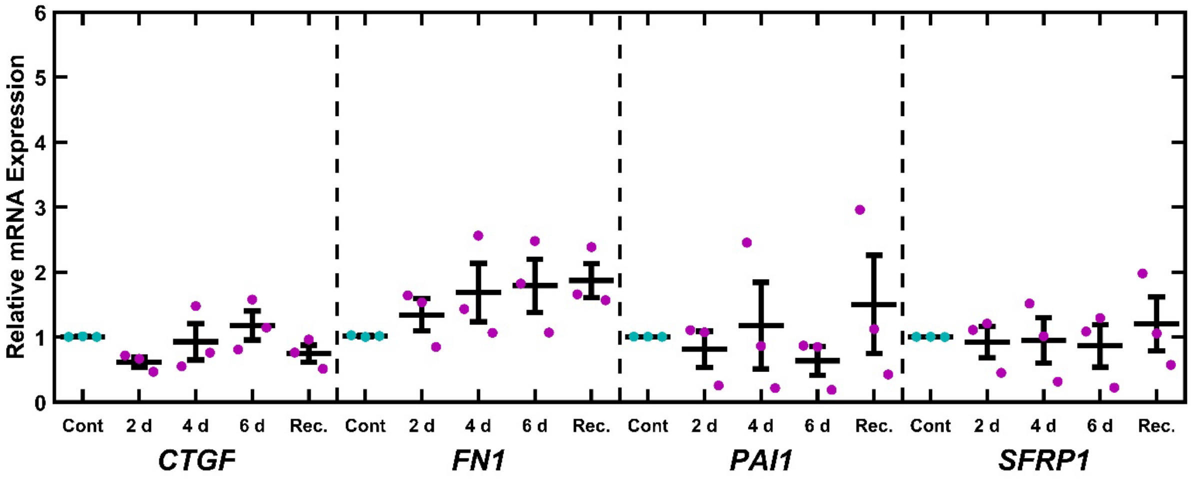

Figure S6: Glaucoma-related mRNA transcripts in untransduced TM-1 cells treated with Dox. After 6 d Dox induction, TM-1 cells showed no significant changes in *CTGF*, *FN1*, *PAI1* and *SFRP1* expression. Magenta/cyan ● represent individual Control/Dox experiments, respectively; mean and standard deviation error bars are represented by black lines. Control data normalized to 1 is shown for reference. Significance in reference to 6 d control was assessed by ANOVA followed by Dunnett’s post-hoc test.

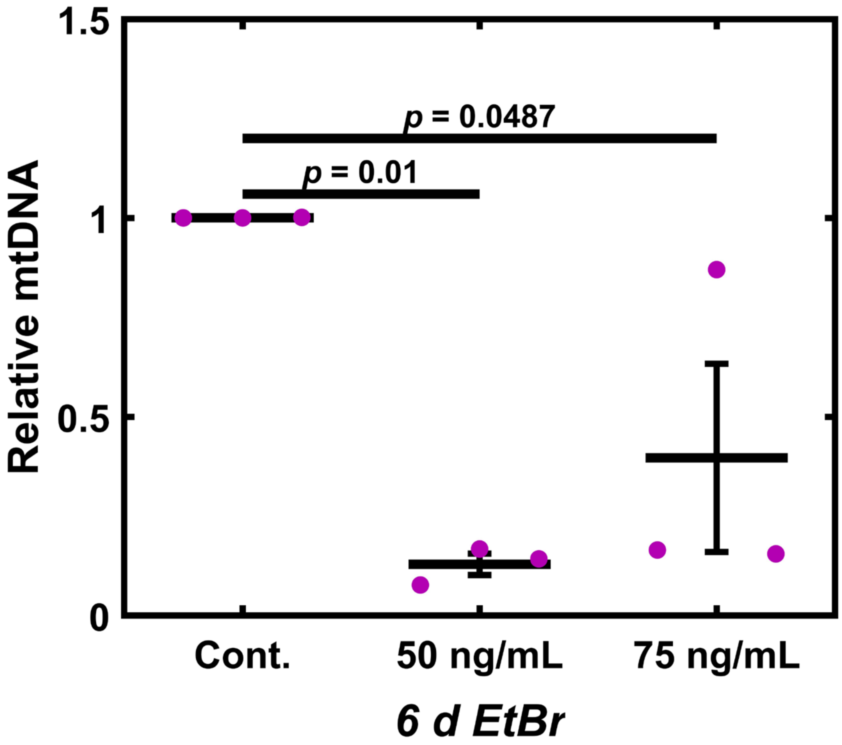

Figure S7: EtBr depletes mtDNA in TM-1 cells. Treatment with 50 or 75 ng/mL EtBr for 6 d resulted significant decrease in mtDNA levels compared to 6 d control culture. Magenta ● represent individual experiments; mean and standard deviation error bars are represented by black lines. Significance between two groups is indicated by a horizontal line and the stated *p* value, assessed by ANOVA followed by Tukey's HSD post-hoc test.
